# Supplementary material for: PTPσ inhibitors promote hematopoietic stem cell regeneration
Source: Nat Commun. 2019 Aug 14;10:3667. doi: 10.1038/s41467-019-11490-5 (PMC6694155; doi:10.1038/s41467-019-11490-5)
Supplement: Supplementary file 1 — Supplementary Information [file 41467_2019_11490_MOESM1_ESM.pdf]

# PTPσ inhibitors promote hematopoietic stem cell regeneration

Zhang et al.

## Supplementary Information

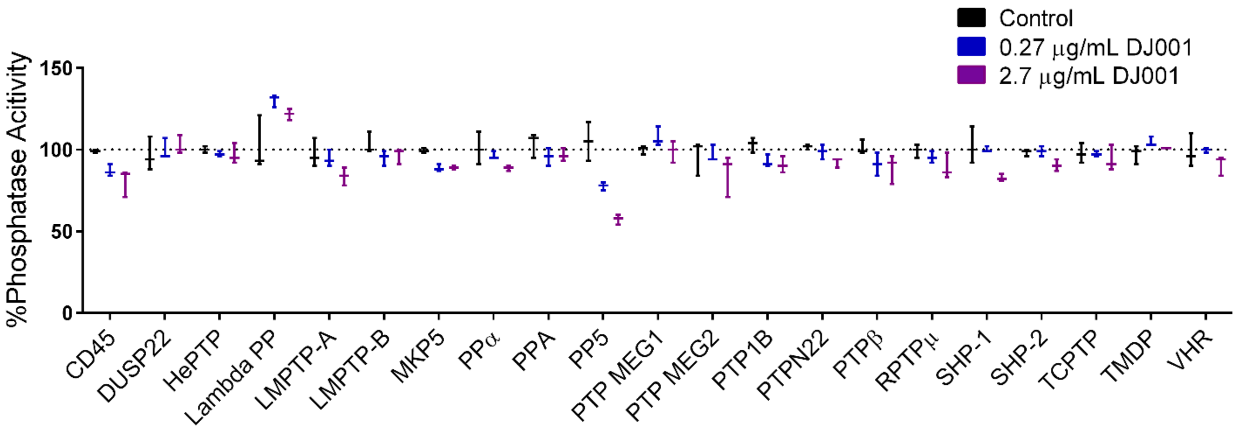


**Supplementary Fig. 1**. Phosphatase inhibitory activity of DJ001. Percent phosphatase activity following exposure to DJ001. CD45: Protein Tyrosine Phosphatase, receptor type C; DUSP22: Dual Specificity Phosphatase 22; HePTP: Haematopoietic Protein Tyrosine Phosphatase; Lambda PP: Lambda Protein Phosphatase; LMPTP-A: Low Molecular Weight Protein Tyrosine Phosphatase A; LMPTP-B: Low Molecular Weight Protein Tyrosine phosphatase B; MKP5: Mitogen-activated Protein Kinase Phosphatase 5; PPα: Protein Phosphatase 1 Catalytic Subunit alpha; PPA: Protein Phosphatase 1; PP5: Protein Phosphatase 5; PTPMEG1: Megakaryocyte Protein-Tyrosine Phosphatase 1; PTPMEG2: Protein Tyrosine Phosphatase Non-Receptor Type 9; PTP1B: Protein Tyrosine Phosphatase 1B; PTPN22: Protein Tyrosine Phosphatase Non-Receptor Type 22; PTPβ: Protein Tyrosine Phosphatase Receptor beta; RPTPμ: Receptor Protein Tyrosine Phosphatase Mu; SHP-1: Protein-Tyrosine Phosphatase 1C; SHP-2: Protein-Tyrosine Phosphatase 2C; TCPTP: T-Cell Protein-Tyrosine Phosphatase; TMDP: Dual Specificity Phosphatase 13; VHR: Dual Specificity Phosphatase 3. Data represent minimum to maximum values (*n* = 3/group). Error bars represent S.E.M.

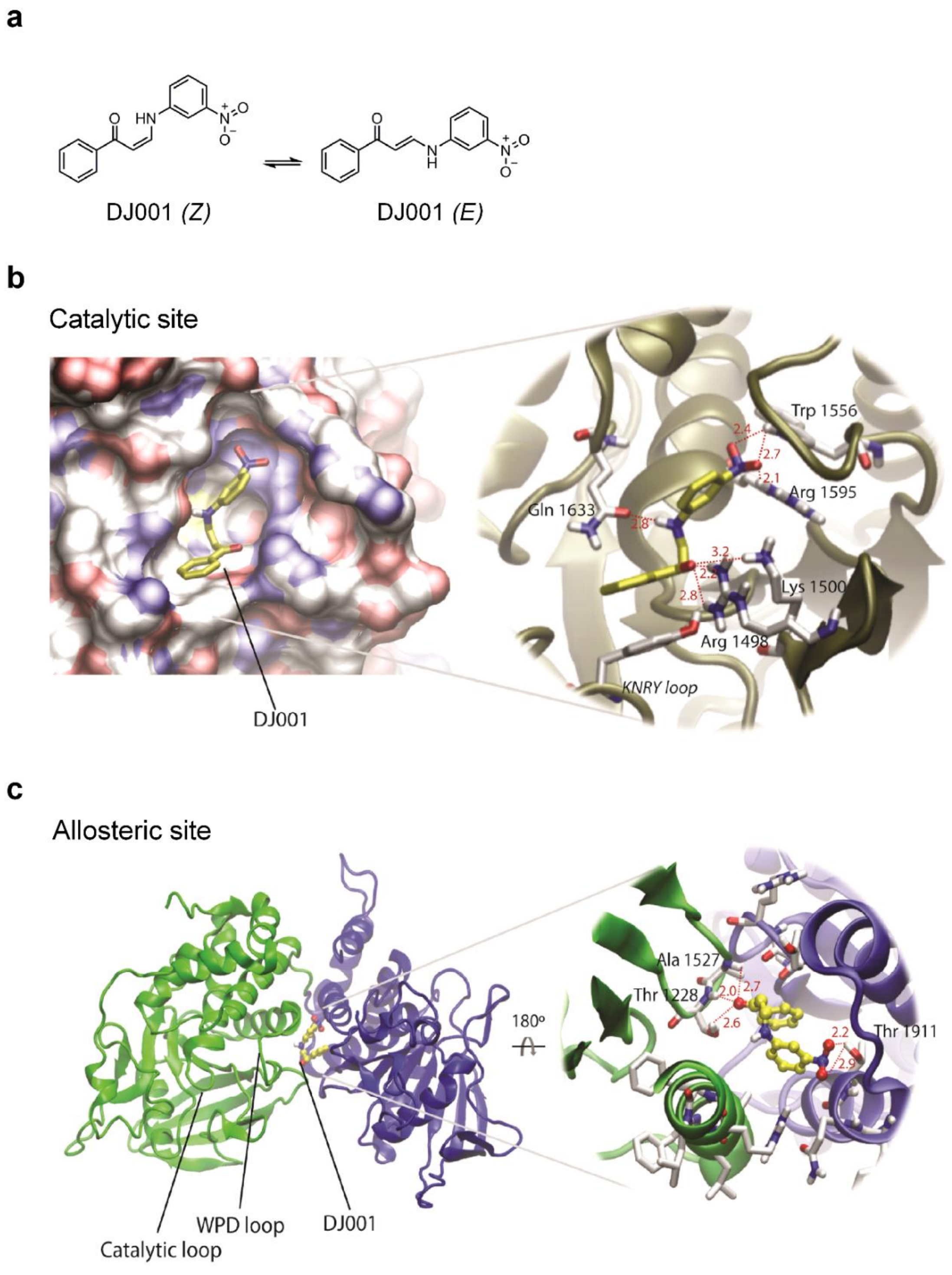


**Supplementary Fig. 2.** DJ001 inhibits PTPσ primarily via allosteric binding. **a** *(Z)* and *(E)*-stereoisomers of DJ001 are present in a 1:1 equilibrium in polar solvents such as DMSO. **b** Details of three dimensional docking of DJ001 *(Z)* to the catalytic binding site of PTPσ (protein data base

ID: 2FH7) which consists of the highly conserved phosphotyrosine recognition loop (KNRY loop: Lysine (K), Asparagine (N), Arginine (R) and Tyrosine (Y)) and important H-bonding interactions to the amino acids, Glutamine (Gln) 1633, L-Tryptophan (Trp) 1556, Arginine (Arg) 1595, Lysine (Lys) 1500 and Arginine (Arg) 1498, shown as red dashed lines with distances between two atoms. Structure of DJ001 *(Z)* represented as capped stick and yellow color. **c** Details of three dimensional docking of DJ001 *(Z)* to the allosteric binding site of human PTPσ (ID: 2FH7) showing important H-bonding interactions to Alanine (Ala) 1527, Threonine (Thr) 1228 and Threonine 1911 and close proximity to the highly conserved Tryptophan (W)/Proline (P)/Aspartate (D) protein loop (WPD loop). Structure of DJ001 *(Z)* is represented as ball and stick in yellow color. H-bonding interactions are shown as red dashed line with distances between two atoms and the catalytic loop as part of the catalytic binding site is indicated in domain 1.

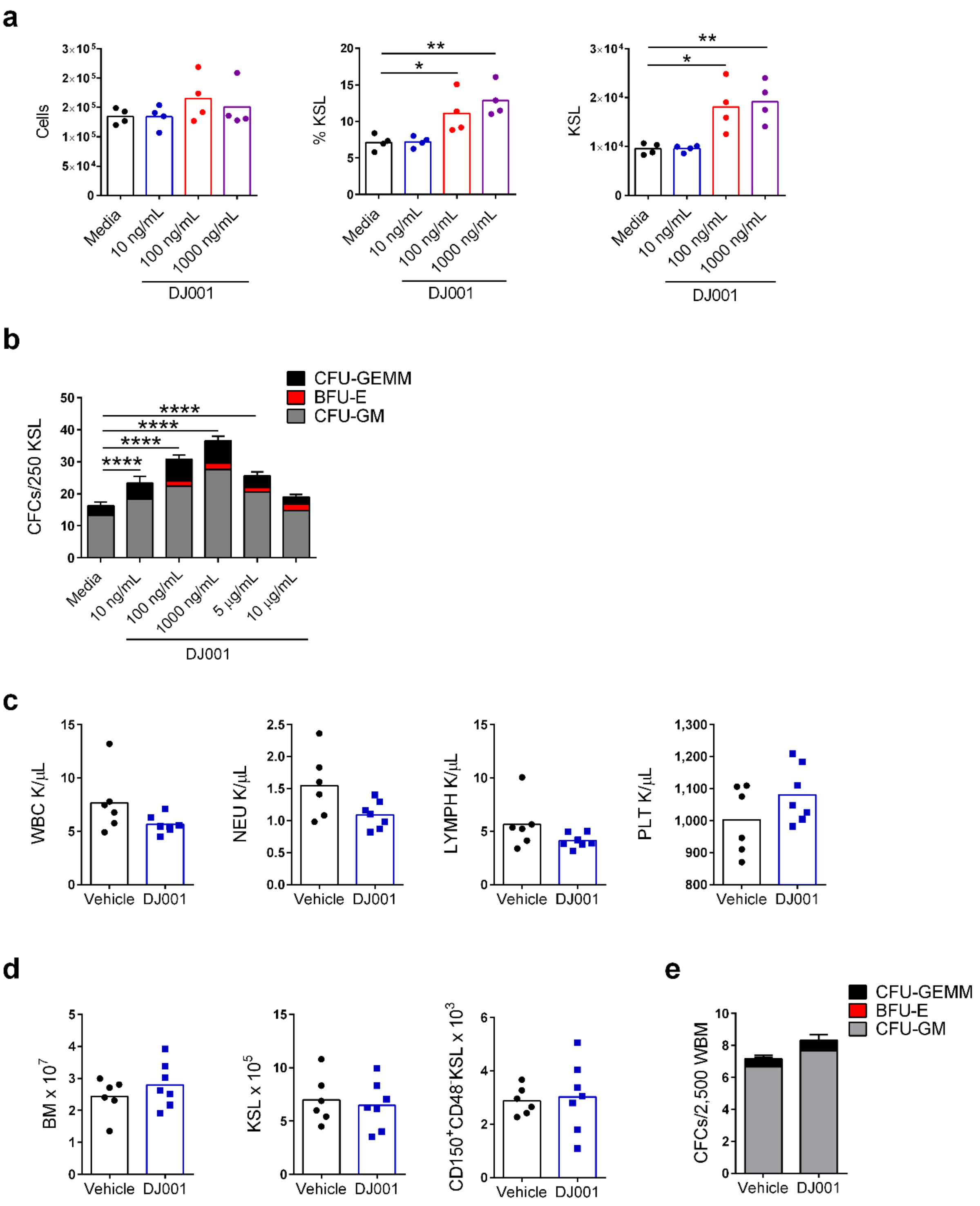


**Supplementary Fig. 3.** DJ001 expands murine BM hematopoietic stem/progenitor cells in culture.

**a** The numbers of cells, percentages of KSL cells and numbers of KSL cells are shown following

culture of BM $CD34^-$KSL cells for 7 days with TSF media (media) + DJ001 (10, 100 or 1,000 ng/ml)(*n* = 4/group). **b** Mean numbers of CFCs at day +3 of culture of BM KSL cells with media + DJ001 (10 ng/mL, 100 ng/mL, 1000 ng/mL, 5 μg/ml or 10 μg/ml)(*n* = 5 - 6/group). **c** Numbers of WBC, NEU, lymphocytes (LYMPH) and platelets (PLT) in the PB of mice treated subcutaneously every M-W-F with 5 mg/kg DJ001 or vehicle for 30 days (vehicle, *n* = 6; DJ001, *n* = 7). **d** BM cell counts and numbers of KSL cells and $CD150^+CD48^-$KSL HSCs in C57BL/6 mice at day +30 following treatment described in **c** (vehicle, *n* = 6; DJ001, *n* = 7). **e** Numbers of CFCs harvested from mice at day +30 following treatment described in **c** (*n* = 6 assays/group). One-way ANOVA with Tukey's multiple comparison test for all comparisons. Error bars represent S.E.M. * $P < 0.05$, ** $P < 0.01$, **** $P < 0.0001$.

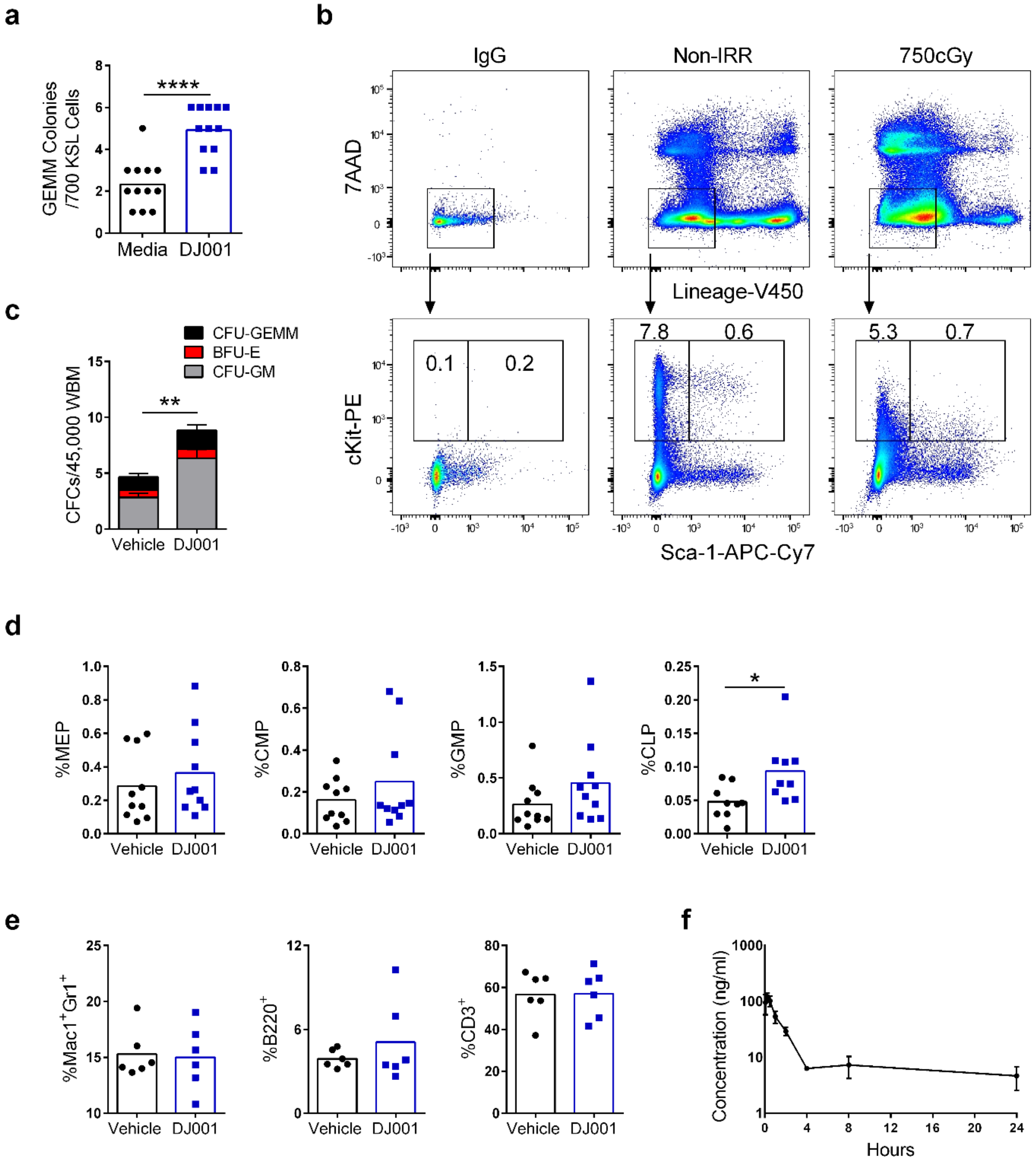


**Supplementary Fig. 4.** Pharmacologic inhibition of PTPσ promotes BM CFC recovery. **a** Mean numbers of CFU-GEMMs within BM KSL cells at day +3 following 300 cGy irradiation and culture in media with or without DJ001 ($n$ = 12/group). **b** Representative gating strategy for BM ckit$^{+}$sca1$^{-}$ lin$^{-}$ cells and KSL cells in IgG stained mouse BM cells, non-irradiated (Non-IRR) mouse BM cells,

and day +10 post 750 cGy irradiated mouse BM cells (750cGy). **c** Numbers of BM CFCs at day +10 post 750 cGy TBI ($n$ = 6). **d** Mean percentages of megakaryocyte-erythrocyte progenitors (MEP), common myeloid progenitors (CMP), granulocyte-monocyte progenitors (GMP), and common lymphoid progenitors (CLP) in the BM at day +10 following 750 cGy irradiation and treatment with DJ001 or vehicle ($n$ = 9 - 10). **e** Mean percentages of PB $Mac1^+Gr1^+$ cells, $B220^+$ cells and $CD3^+$ cells in mice at day +10 following 750 cGy TBI and treatment with DJ001 or vehicle ($n$ = 6). **f** Pharmacokinetics (PK) for mean plasma concentrations of DJ001 in adult C57BL/6 mice following subcutaneous administration of 5 mg/kg DJ001 ($n$ = 3). Error bars represent S.E.M. * $P < 0.05$, ** $P < 0.01$, **** $P < 0.0001$.

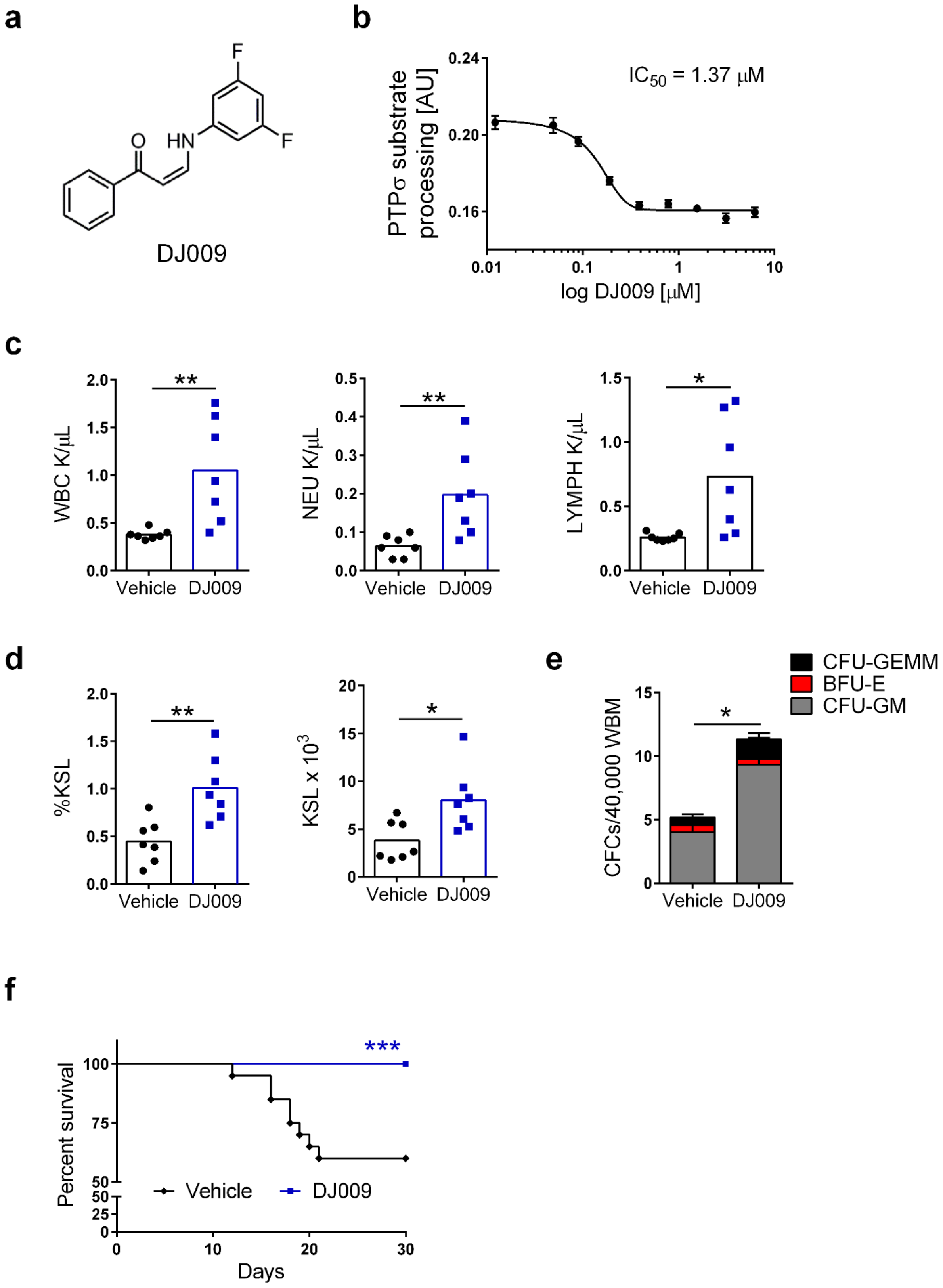

a
DJ009
b
IC50 = 1.37 μM
PTPσ substrate processing [AU]
log DJ009 [μM]
c
WBC K/μL
NEU K/μL
LYMPH K/μL
Vehicle
DJ009
d
%KSL
KSL x 10^3
e
CFU-GEMM
BFU-E
CFU-GM
CFCs/40,000 WBM
f
Percent survival
Days
Vehicle
DJ009

**Supplementary Fig. 5.** DJ009 inhibits PTPσ and promotes hematopoietic regeneration. **a** Chemical structure for compound DJ009. **b** Concentration-inhibition curves and $IC_{50}$ value for DJ009. Data points are means of absorption unit (*n* = 3). **c** Mean numbers of PB WBCs, NEU, and LYMPH in mice at day +10 following 750 cGy and treatment with 5 mg/kg DJ009 or vehicle (*n* = 7). **d** Mean percentages and numbers of BM KSL cells at day +10 post 750 cGy followed by treatment with DJ009 or vehicle (*n* = 7). **e** Mean numbers of BM CFCs at day +10 post 750 cGy TBI and treatment with DJ009 or vehicle (*n* = 9-10/group). **f** Survival of mice irradiated with 750 cGy and treated with 5 mg/kg DJ009 (24/24 mice) or vehicle (12/20 mice) daily for 10 d (*** $P$ = 0.0006, log-rank test). Error bars represent S.E.M. * $P < 0.05$, ** $P < 0.01$, *** $P < 0.001$.

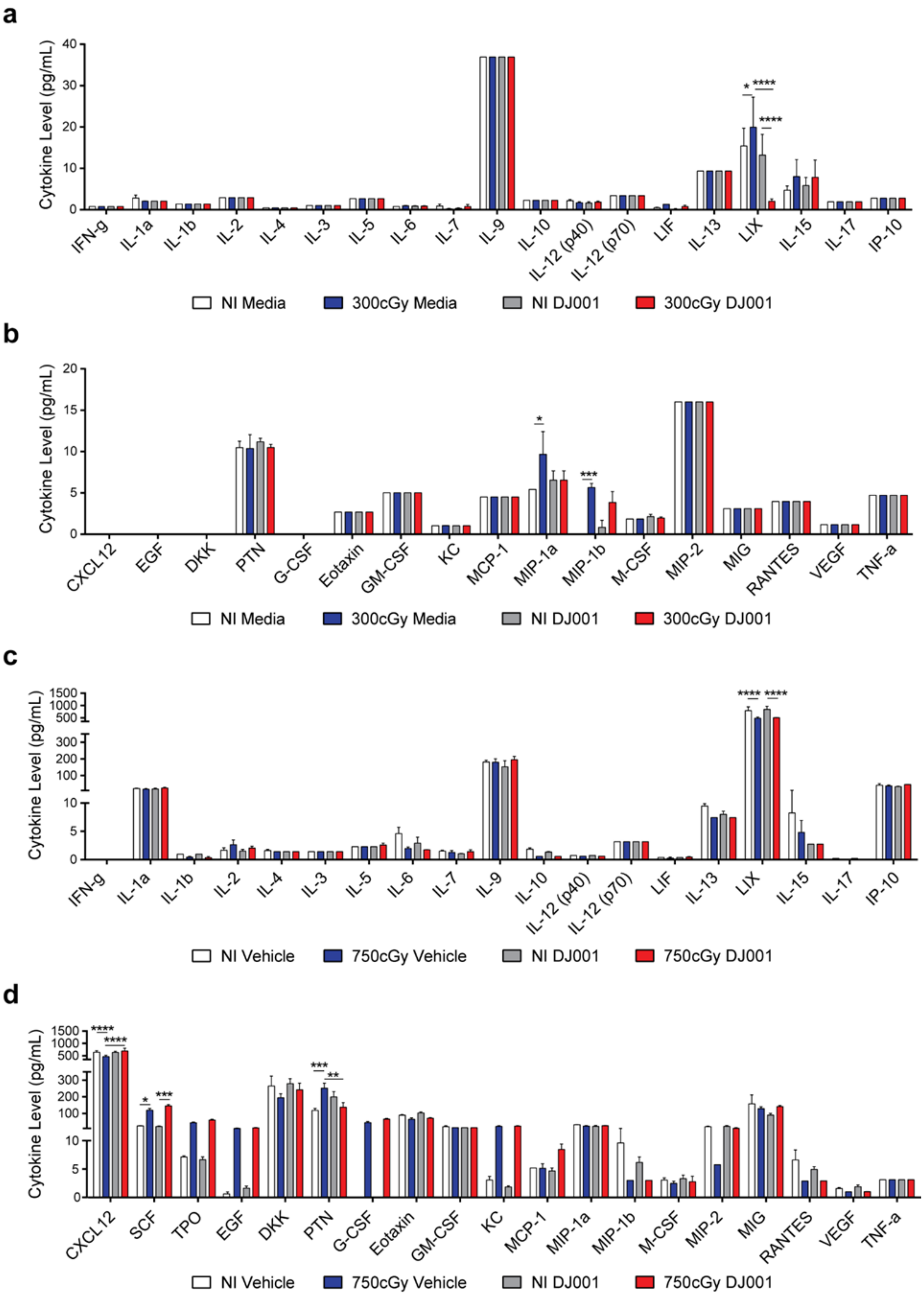
a
Cytokine Level (pg/mL)
IFN-g
IL-1a
IL-1b
IL-2
IL-4
IL-3
IL-5
IL-6
IL-7
IL-9
IL-10
IL-12 (p40)
IL-12 (p70)
LIF
IL-13
LIX
IL-15
IL-17
IP-10
NI Media
300cGy Media
NI DJ001
300cGy DJ001
b
Cytokine Level (pg/mL)
CXCL12
EGF
DKK
PTN
G-CSF
Eotaxin
GM-CSF
KC
MCP-1
MIP-1a
MIP-1b
M-CSF
MIP-2
MIG
RANTES
VEGF
TNF-a
NI Media
300cGy Media
NI DJ001
300cGy DJ001
c
Cytokine Level (pg/mL)
IFN-g
IL-1a
IL-1b
IL-2
IL-4
IL-3
IL-5
IL-6
IL-7
IL-9
IL-10
IL-12 (p40)
IL-12 (p70)
LIF
IL-13
LIX
IL-15
IL-17
IP-10
NI Vehicle
750cGy Vehicle
NI DJ001
750cGy DJ001
d
Cytokine Level (pg/mL)
CXCL12
SCF
TPO
EGF
DKK
PTN
G-CSF
Eotaxin
GM-CSF
KC
MCP-1
MIP-1a
MIP-1b
M-CSF
MIP-2
MIG
RANTES
VEGF
TNF-a
NI Vehicle
750cGy Vehicle
NI DJ001
750cGy DJ001

**Supplementary Fig. 6.** Effects of irradiation and DJ001 on cytokine levels in vitro and in vivo. **a** In vitro levels of interleukins and IFN-gamma at 12 hours of culture of non-irradiated (white bar) and irradiated (300 cGy, blue bar) KSL cells, treated with and without 1 µg/ml DJ001 (gray bar and red bar, respectively)(*n* = 3/group). **b** In vitro levels of HSC growth factors and other cytokines at 12 hours of culture of the populations described in **a**. SCF and TPO not shown since part of TSF media. **c** In vivo levels of interleukins and IFN-gamma in the BM of non-irradiated (white bar) and 750 cGy – irradiated mice (blue bar) at day +5 of vehicle treatment; cytokine levels also shown in the same mice groups treated for 5 days with 5 mg/kg DJ001 (gray and red bar, respectively)(*n* = 3/group). **d** Levels of HSC growth factors and other cytokines at day +5 in the BM of the mice described in **c**. 2-way ANOVA with Sidak's multiple comparison test was performed for all comparisons. IFN-g, Interferon gamma; IL-1a, Interleukin 1 alpha; IL-1b, Interleukin 1 beta; IL-2, Interleukin 2; IL-3, Interleukin 3; IL-4, Interleukin 4; IL-5, Interleukin 5; IL-6, Interleukin 6; IL-7, Interleukin 7; IL-9, Interleukin 9; IL-10, Interleukin 10; IL-12 p40, Interleukin 12 subunit p40; IL-12 p70, Interleukin 12 subunit 70; LIF, Leukemia Inhibitory Factor; IL-13, Interleukin 13; LIX, LPS-induced C-X-C Chemokine; IL-15, Interleukin 15; IL-17, Interleukin 17; IP-10, Interferon gamma induced protein 10; CXCL12, C-X-C Motif Chemokine Ligand 12; EGF, Epidermal Growth Factor; DKK1, Dickkopf 1; PTN, Pleiotrophin; G-CSF, Granulocyte Colony Stimulating Factor; GM-CSF, Granulocyte-Macrophage Colony Stimulating Factor; KC, C-X-C motif ligand 1; MCP-1a, Monocyte Chemoattractant Protein-1; MIP-1a, Macrophage Inflammatory Protein -1 alpha; MIP-1b, Macrophage Inflammatory Protein -1 beta; M-CSF, Macrophage Colony Stimulating Factor; MIP-2, Macrophage Inflammatory Protein - 2; MIG, Monokine Induced by Gamma Interferon; RANTES, Regulated on Activation, Normal T cell Expressed and Secreted protein; VEGF, Vascular Endothelial Growth Factor; TNF-a, Tumor Necrosis Factor Alpha. Error bars represent S.E.M. * $P < 0.05$, ** $P < 0.01$, *** $P < 0.001$, **** $P < 0.0001$.

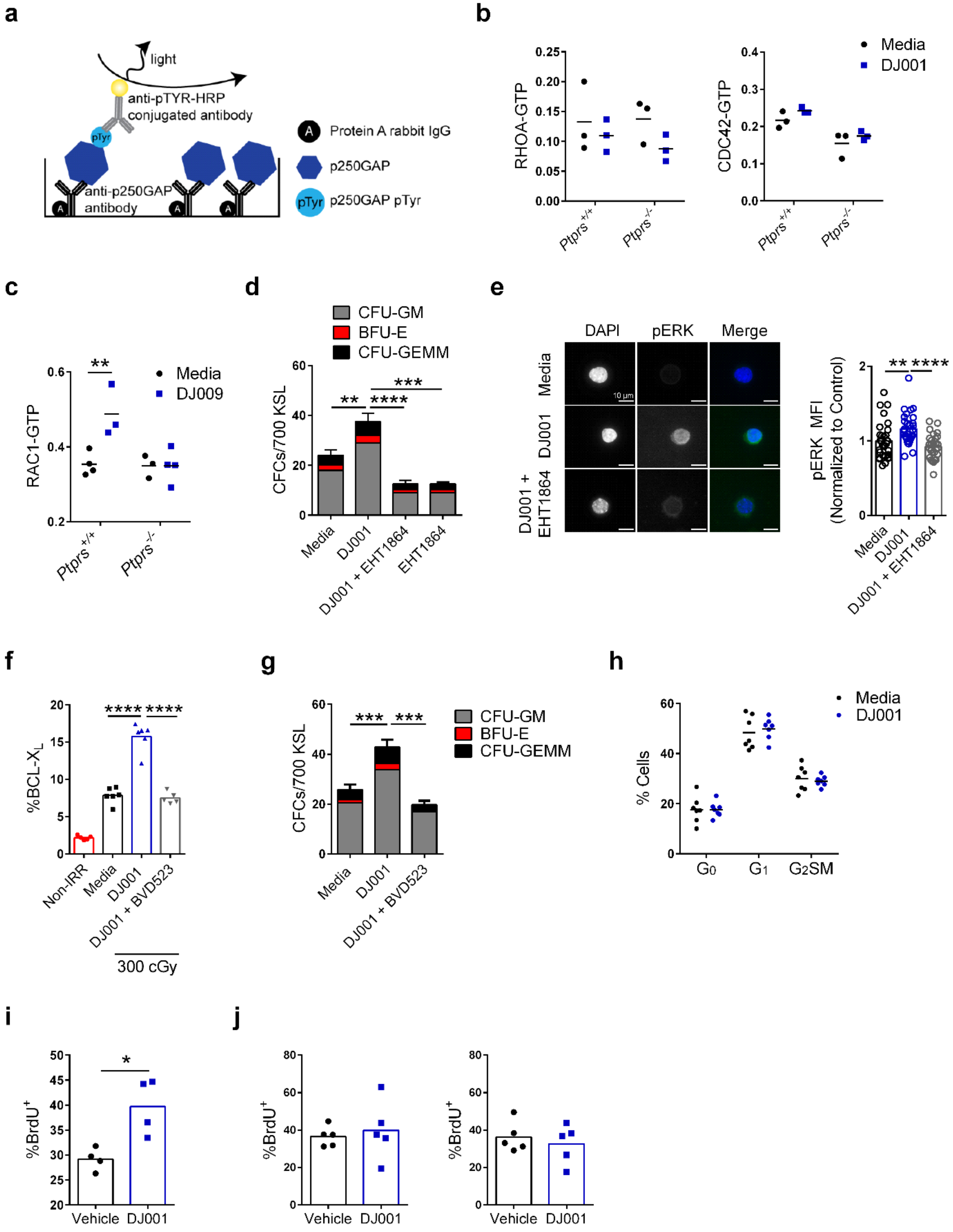
a
light
anti-pTYR-HRP conjugated antibody
pTyr
anti-p250GAP antibody
A
Protein A rabbit IgG
p250GAP
p250GAP pTyr
b
RHOA-GTP
CDC42-GTP
Ptprs+/+
Ptprs-/-
Media
DJ001
c
RAC1-GTP
Media
DJ009
d
CFU-GM
BFU-E
CFU-GEMM
CFCs/700 KSL
Media
DJ001
DJ001 + EHT1864
EHT1864
e
DAPI
pERK
Merge
Media
DJ001
DJ001 + EHT1864
10 µm
pERK MFI (Normalized to Control)
f
%BCL-XL
Non-IRR
Media
DJ001
DJ001 + BVD523
300 cGy
g
CFCs/700 KSL
CFU-GM
BFU-E
CFU-GEMM
h
% Cells
Media
DJ001
G0
G1
G2SM
i
%BrdU+
Vehicle
DJ001
j
%BrdU+
Vehicle
DJ001

**Supplementary Fig. 7.** DJ001 promotes HSPC regeneration via RAC pathway activation. **a** Schematic of p250GAP phospho-tyrosine (pTyr) sandwich ELISA assay. **b** Mean RHOA-GTP and CDC42-GTP levels in BM lin$^-$ cells from *Ptprs*$^{+/+}$ and *Ptprs*$^{-/-}$ mice that were pulse-treated in media $\pm$ 1 µg/ml DJ001 for 10 min (*n* = 3/group). **c** Mean RAC1-GTP levels in BM lin$^-$ cells from *Ptprs*$^{+/+}$ mice and *Ptprs*$^{-/-}$ mice that were pulse-treated in media $\pm$ 1 µg/ml DJ009 for 10 min. Two-way ANOVA with Sidak's multiple comparison test (*n* = 3 - 4/group). **d** Numbers of BM CFCs at day +3 from BM KSL cells irradiated with 300 cGy and cultured with media $\pm$ 1 µg/ml DJ001, DJ001 + 6 µg/ml EHT1864 and EHT1864 alone (*n* = 6-12/group). **e** At left, microscopic images of BM KSL cells cultured in media $\pm$ DJ001, or DJ001 + 6 µg/ml EHT1864 for 10 minutes, and stained for pERK and DAPI (scale bar = 10 µm). At right, MFI of pERK. One-way ANOVA with Tukey's multiple comparison test. **f** Percentages of BCL-$X_L$ protein in non-irradiated (non-IRR) BM KSL cells and at 24 hours following 300 cGy and culture with media $\pm$ DJ001, or media $\pm$ DJ001 + 43 ng/ml BVD523 (*n* = 5 - 6/group). One-way ANOVA with Tukey's multiple comparison test. **g** Numbers of BM CFCs at day +3 of culture of BM KSL cells following 300 cGy and treatment with media $\pm$ DJ001, or DJ001 + 43 ng/mL BVD523 (*n* = 5 - 12 assays/group). One-way ANOVA with Tukey's multiple comparison test. **h** Percentages of BM KSL cells in $G_0$, $G_1$, and $G_2$/S/M phases after culture x 36 hours in media $\pm$ DJ001 (*n* = 7/group). **i** %BrdU$^+$KSL cells at day +10 in mice following 750 cGy TBI and treated daily with 5 mg/kg DJ001 or vehicle (*n* = 4). **j** %BrdU$^+$CD45.2$^+$ donor cells at day +7 and day +21 following competitive transplantation into CD45.1$^+$ recipient mice, as described in Figure 2 (*n* = 5). Error bars represent S.E.M. * $P < 0.05$, ** $P < 0.01$, *** $P < 0.001$, **** $P < 0.0001$.

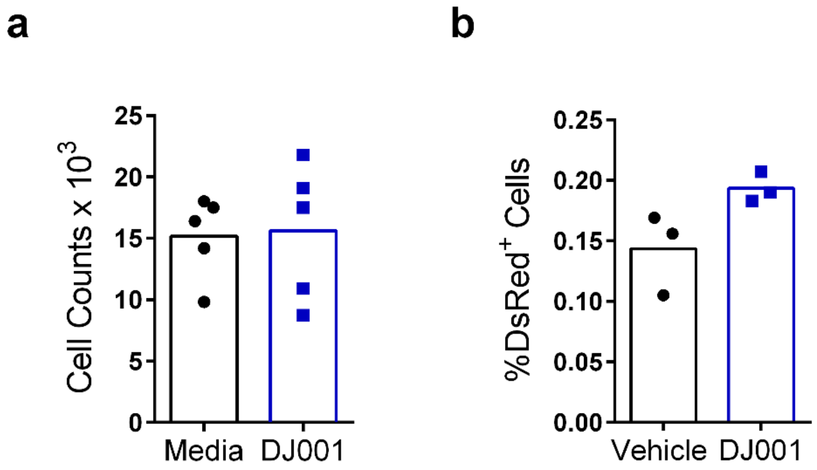


**Supplementary Fig. 8.** Effect of DJ001 treatment on BM HSPC migration and homing. **a** Mean number of cells within the lower chambers of a trans-endothelial migration assay at 18 hours following culture of BM KSL cells in the upper chambers coated with mouse spleen ECs and treatment with and without 1 µg/ml DJ001 (*n* = 5/group). **b** Mean percentage of donor $DsRed^+$ cells in the BM of mice at 18 hours following intravenous injection of 1 x $10^5$ $DsRed^+Sca1^+Lin^-$ cells and treatment with 5 mg/kg DJ001 or vehicle subcutaneously (*n* = 3/group).
