# Supplementary material for: PTPσ inhibitors promote hematopoietic stem cell regeneration
Source: Nat Commun. 2019 Aug 14;10:3667. doi: 10.1038/s41467-019-11490-5 (PMC6694155; doi:10.1038/s41467-019-11490-5)
Supplement: Supplementary file 3 — Reporting Summary [file 41467_2019_11490_MOESM3_ESM.pdf]

nature research

Corresponding author(s): John P. Chute

Last updated by author(s): May 9, 2019

Reporting Summary

Nature Research wishes to improve the reproducibility of the work that we publish. This form provides structure for consistency and transparency in reporting. For further information on Nature Research policies, see Authors & Referees and the Editorial Policy Checklist.

Statistics

For all statistical analyses, confirm that the following items are present in the figure legend, table legend, main text, or Methods section.

n/a	Confirmed	
☐	☒	The exact sample size (*n*) for each experimental group/condition, given as a discrete number and unit of measurement
☐	☒	A statement on whether measurements were taken from distinct samples or whether the same sample was measured repeatedly
☐	☒	The statistical test(s) used AND whether they are one- or two-sided<br>*Only common tests should be described solely by name; describe more complex techniques in the Methods section.*
☐	☒	A description of all covariates tested
☐	☒	A description of any assumptions or corrections, such as tests of normality and adjustment for multiple comparisons
☐	☒	A full description of the statistical parameters including central tendency (e.g. means) or other basic estimates (e.g. regression coefficient) AND variation (e.g. standard deviation) or associated estimates of uncertainty (e.g. confidence intervals)
☐	☒	For null hypothesis testing, the test statistic (e.g. *F*, *t*, *r*) with confidence intervals, effect sizes, degrees of freedom and *P* value noted<br>*Give P values as exact values whenever suitable.*
☒	☐	For Bayesian analysis, information on the choice of priors and Markov chain Monte Carlo settings
☒	☐	For hierarchical and complex designs, identification of the appropriate level for tests and full reporting of outcomes
☐	☒	Estimates of effect sizes (e.g. Cohen's *d*, Pearson's *r*), indicating how they were calculated

Our web collection on statistics for biologists contains articles on many of the points above.

Software and code

Policy information about availability of computer code

Data collection	Immunofluorescent pictures were collected using ZEISS ZEN Software (Microscopy), Flow Cytometry results were acquired using BD FACSDIVA (BD Biosciences, NJ), Tecan Sparkcontrol (plate reader assays). Docking studies were performed using AutoDock Vina (Scripps Research Institute, CA) and Maestro 10.5 (Schrödinger LLC, NY).
Data analysis	All statistical analyses were performed using GraphPad PRISM v6 (La Jolla, CA), Immunofluorescent pictures were analyzed using Fuji (ImageJ), Flow Cytometry analyses were performed using BD FACSDIVA (BD Biosciences, NJ) and FlowJo v10 (Treestar, Ashland, OR). Docking studies were analyzed with PyMOL (Schroedinger LLC, NY) and visualized by VMD 1.9.2 (UIUC, IL).

For manuscripts utilizing custom algorithms or software that are central to the research but not yet described in published literature, software must be made available to editors/reviewers. We strongly encourage code deposition in a community repository (e.g. GitHub). See the Nature Research guidelines for submitting code & software for further information.

Data

Policy information about availability of data

All manuscripts must include a data availability statement. This statement should provide the following information, where applicable:

- Accession codes, unique identifiers, or web links for publicly available datasets
- A list of figures that have associated raw data
- A description of any restrictions on data availability

Complete datas are provided as a Source Data file.

Field-specific reporting

Please select the one below that is the best fit for your research. If you are not sure, read the appropriate sections before making your selection.

☒ Life sciences ☐ Behavioural & social sciences ☐ Ecological, evolutionary & environmental sciences

For a reference copy of the document with all sections, see nature.com/documents/nr-reporting-summary-flat.pdf

Life sciences study design

All studies must disclose on these points even when the disclosure is negative.

Sample size	For all animal survival studies a sample size of n =14 - 15 mice was used to achieve a 95% power to detect a mean difference of 1 standard deviation with a significance level of 0.05 using a Log-rank (Mantel-Cox) test. Competitive repopulation assays were performed with a sample size of n=13 - 14 mice and hematopoietic recovery studies were performed with n = 8 - 9 to achieve a 95% power to detect a mean difference of 1 standard deviation with a significance level of 0.05 using a two-tailed student t-test. Sample sizes were also contributed to by independent replicate experiments.
Data exclusions	No data were excluded from the analysis.
Replication	All attempts at replication were successful.
Randomization	All mice used in this study were weight, sex and age matched prior to their allocation into treatment groups.
Blinding	No blinding was performed.

Reporting for specific materials, systems and methods

We require information from authors about some types of materials, experimental systems and methods used in many studies. Here, indicate whether each material, system or method listed is relevant to your study. If you are not sure if a list item applies to your research, read the appropriate section before selecting a response.

Materials & experimental systems

n/a	Involved in the study	
☐	☒	Antibodies
☒	☐	Eukaryotic cell lines
☒	☐	Palaeontology
☐	☒	Animals and other organisms
☒	☐	Human research participants
☒	☐	Clinical data

Methods

n/a	Involved in the study	
☒	☐	ChIP-seq
☐	☒	Flow cytometry
☒	☐	MRI-based neuroimaging

Antibodies

Antibodies used	Antibodies used in these studies were: anti-c-kit phycoerythrin (PE)-Cy7 (BD Biosciences), V450 lineage cocktail (BD Biosciences), Alexa Fluor 488-conjugated anti-CD48 (BioLegend), Alexa Fluor 647-conjugated anti-CD150 (Biolegend), BV605 anti-CD45.2 (BioLegend), fluorescein isothiocyanate (FITC)-conjugated anti-CD45.1 (BD Biosciences), PE-conjugated anti-Mac-1 and anti-Gr-1 (BD Biosciences), APC-conjugated anti-Ter119 (BD Biosciences), V450-conjugated anti-CD3 (BD Biosciences), and APC-Cy7-conjugated anti-B220 (BD Biosciences), FITC-conjugated anti-Bcl-xL (Abcam #ab26148), FITC-conjugated anti-Mcl1 (Abcam #197529), and anti-PAK1 (phospho S144) + PAK2 (phospho S141) + PAK3 (phospho S139) antibody (Abcam #ab40795).
Validation	All antibodies used are validated for detection of indicated proteins

Animals and other organisms

Policy information about studies involving animals; ARRIVE guidelines recommended for reporting animal research

Laboratory animals	In this study we used mice bearing constitutive deletion of Ptprs (Ptprs -/-) in a Balb/c background (female and male, 8-12 weeks), C57BL/6 mice (female and male, 8-12 weeks), B6.SJL and NOD.Cg-Prkdc scid Il2rg tm1Wjl/ SzJ (NSG) mice (female and male, 8-12 weeks).
Wild animals	n/a
Field-collected samples	n/a

Ethics oversight	All animal studies were approved by UCLA Animal Research Committee, Approval Number 2014-021-13M.

Note that full information on the approval of the study protocol must also be provided in the manuscript.

Flow Cytometry

Plots

Confirm that:

☒ The axis labels state the marker and fluorochrome used (e.g. CD4-FITC).

☒ The axis scales are clearly visible. Include numbers along axes only for bottom left plot of group (a 'group' is an analysis of identical markers).

☒ All plots are contour plots with outliers or pseudocolor plots.

☒ A numerical value for number of cells or percentage (with statistics) is provided.

Methodology

Sample preparation	Detailed sample preparation and source of samples are listed in Methods.
Instrument	BD FACS Canto II and FACS Aria II
Software	BD FACSDIVA (BD Biosciences, NJ) for collection and FlowJo v10 (Treestar, Ashland, OR) for analysis.
Cell population abundance	KSLs were sorted per mouse and purity was assessed by staining for ckit+ sca1+ Lin- CD34+ and CD150+
Gating strategy	Relevant gating strategies shown in corresponding flow plots and Supplementary Information.

☒ Tick this box to confirm that a figure exemplifying the gating strategy is provided in the Supplementary Information.
